# Research and education in analytical chemistry — industrial and academic perspectives from a survey conducted in Sweden

**DOI:** 10.1007/s00216-023-04661-3

**Published:** 2023-03-30

**Authors:** Jonas Bergquist, Åsa Emmer, Anne Farbrot, Charlotta Turner

**Affiliations:** 1 Department of Chemistry – BMC, Analytical Chemistry, Uppsala University, Box 599, SE-75124 Uppsala, Sweden; 2 Analytical Chemistry, Division of Applied Physical Chemistry, Department of Chemistry, KTH Royal Institute of Technology, Teknikringen 36, SE-10044 Stockholm, Sweden; 3 Department of Occupational and Environmental Health, Sahlgren’s University Hospital, P.O. Box 414, SE-40530 Gothenburg, Sweden; 4 Department of Chemistry, Centre for Analysis and Synthesis, Lund University, P.O. Box 124, SE-22100 Lund, Sweden

## Introduction

Analytical chemistry is a sub-discipline of chemistry that even young schoolchildren encounter at an early stage in their education. Gravimetry and measurement uncertainties are aspects that form a part of such education, as well as measurements of pH and colorimetry. These are all an integral part of children’s early educational experiences, which shows the importance of this discipline within the wider context of natural science.

In Sweden, there are 16 universities (not counting university colleges), all of which offer courses or classes in analytical chemistry. There is also a longstanding and strong industry in pharmaceutical and fine chemical research and development, where competence in analytical chemistry is essential. There has been a growing concern that the subject of analytical chemistry is losing ground to other disciplines, or rather, losing ground without any other discipline filling the gaps. Many universities, not only in Sweden, have stopped giving or substantially reduced giving courses in analytical chemistry in relation to other subjects, e.g. from 43.5 ECTS in 2010 to 32 ECTS in 2022 at KTH Royal Institute of Technology, and from 75 ECTS in 2007 to 30 ECTS in 2022 at Lund University, Faculty of Science (Faculty of Engineering; from 22.5 to 15 ECTS during the same time period). (ECTS, The European Credit Transfer and Accumulation System). It has in parallel become increasingly difficult for industry to recruit competent analytical chemists [1, 2]. For university researchers, it has become harder to obtain external funding for fundamental research in analytical chemistry, and one often heard opinion is that analytical chemistry is considered mainly as a “tool” for other disciplines. A concept best described as “analytics” is taking shape, meaning that chemical analysis is becoming a “black box” operation — sample in, data out. Perhaps popular crime series have perpetuated the idea of analytical chemistry being a method-only discipline and that it should be quick and easy. These assumptions of analytical chemistry as a ready-to-use tool may be further strengthened by the use of advanced chemical analyses in other disciplines, which is natural and once again emphasises that the analytical chemistry of today is very different from what it used to be.

The Analytical Chemistry Division of the Swedish Chemical Society has initiated a think tank focused on “what analytical chemistry is”, and whether this has changed over the years, or at least if scientists outside the field have a different opinion on this matter than the analytical chemists themselves. The question is: who are the analytical chemists in Sweden, and what are our work tasks as analytical chemists? What type of analytical chemistry do we teach, and does this correspond well with industry demand? Is the subject and definition of analytical chemistry changing with time? Is this change unavoidable and necessary for the adaption to society in general and the scientific map specifically? How can we best collaborate with other disciplines?

This article presents the outcome of a survey conducted in Sweden, targeting especially the analytical chemists at universities, government institutes, and industry, as well as other non-analytical chemists.

## Information about the survey and the participants

**Fig. 1:**
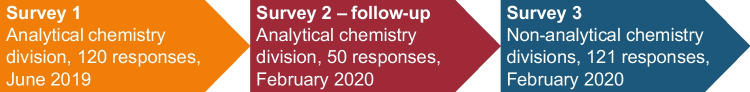
Surveys conducted by the Analytical Chemistry Division of the Swedish Chemical Society during 2019 and 2020

The process of the surveys is illustrated in Fig. 1. The response rate of the first survey was 120 out of 360, i.e. 33%, and it was targeting members of the Analytical Chemistry Division of the Swedish Chemical Society. The follow-up survey was sent to the same group and had a response rate of 50 out of 360, i.e. 14%, while survey 3 targeted chemists in all other divisions of the Swedish Chemical Society. Here the response rate was merely 121 out of ca. 2000, i.e. 6%. The surveys were done using SurveyMonkey®, and all answers are anonymous. Post-editing was only done to remove obvious duplicates. Data is presented as “all” (includes all responses), “university” (those affiliated with a university), and “industry” (those employed by an industry). Some answers were from “research institute”, “retired”, and “other”. These are included in the “all” category. All raw data from the surveys are available upon request, either from the authors or from the Swedish Chemical Society.

## Who are the analytical chemists in Sweden?

**Fig. 2:**
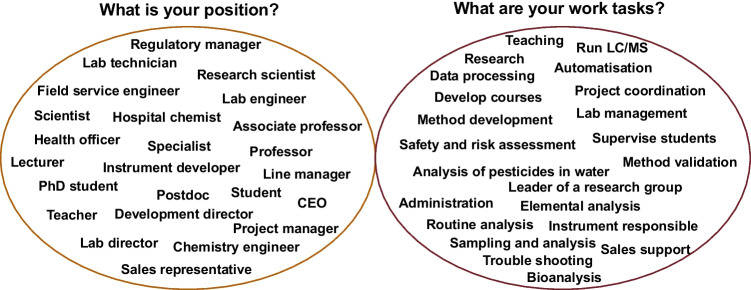
A selection of free-text answers to the questions “What is your position?” (left), and “What are your work tasks?” (right)

There is obviously no single description that fits all analytical chemists. The survey reveals that Swedish analytical chemists work in different environments, and with a large variety of work tasks (Fig. 2). The diversity is evidently large, but some conclusions can still be drawn from the cohort responding to the first two surveys.

**Fig. 3:**
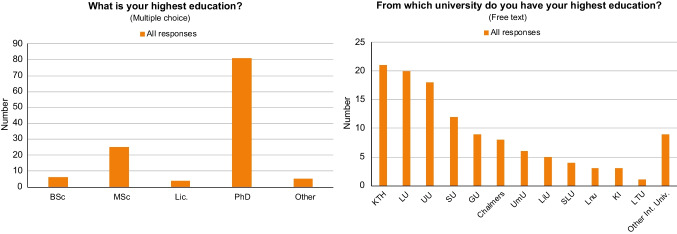
Responses in the first survey to the questions “What is your highest education?” (left), and “From which university do you have your highest education?” (right). Lic., Licentiate degree (ca. “half of a PhD degree”); KTH, The Royal Institute of Technology; LU, Lund University; UU, Uppsala University; SU, Stockholm University; GU, Gothenburg University; Chalmers, Chalmers University of Technology; UmU, Umeå University; LiU, Linköping University; SLU, Swedish University of Agricultural Sciences; Lnu, Linnaeus University, KI, Karolinska Institutet; LTU, Luleå University of Technology. Other international universities: Johns Hopkins University, Indian Institute of Science Education and Research, Leipzig University, Louis Pasteur University, Copenhagen University, Tuzla University, Wageningen Agricultural University, Saint Petersburg State University, and Åbo Academy University

The responders are mainly working in either industry (40% survey 1, 25% survey 2) or academia (35% survey 1, 40% survey 2). The most common academic education level is doctoral studies (50% survey 1, 70% survey 2). Nonetheless, in survey 1, 25% hold a master’s degree, and the remaining respondents are primarily made up of licentiate degrees (approximately half of a doctoral degree) and bachelor degrees (Fig. 3). The year of examination ranges from 1967 to 2019, with a majority from 1993 and later.

The degrees have been issued from 20 different universities, of which 14 are Swedish institutions (survey 1, fewer in survey 2). The four most common universities are Stockholm University, Uppsala University, Lund University, and Gothenburg University. In survey 2, the KTH Royal Institute of Technology is also one of the most frequently mentioned universities. The rest of the universities listed are spread all over Sweden (Fig. 3).

Survey 2 included specific questions regarding internationalization, mobility, and collaboration in academic milieus. The answers to the question concerning the presence of international group members ranged between one and ten per research group. Moreover, 43% of the responders have been abroad for more than 3 months (post-doctoral stays and research visits etc.), and the number of collaboration projects the responders are involved in ranges between 1 and 15 (typically 2–5).

## What do you do as an analytical chemist?

**Fig. 4:**
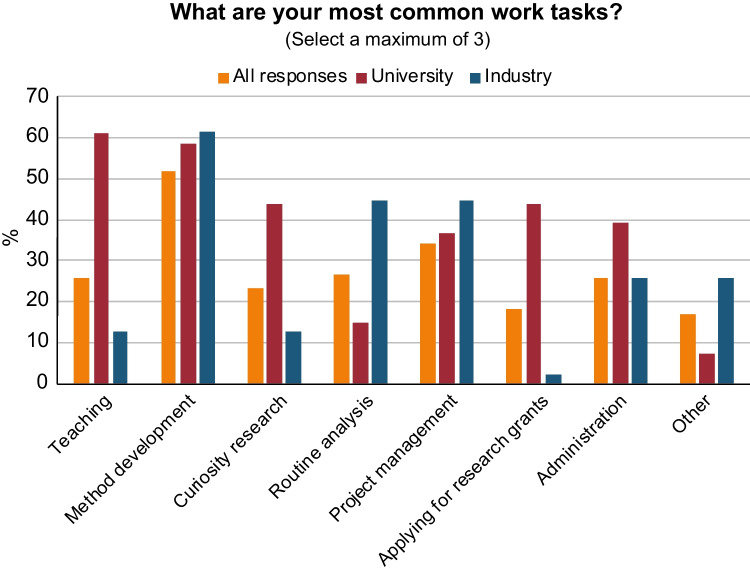
Responses to the question: “What are your most common work tasks?”. “All responses” include persons from industry, university, research institutes, authorities, students, and retired. On the *y*-axis: response in percentage from each sub-category

Analytical chemistry as a subject is often defined as “what analytical chemists do” [3]. What are the work tasks of an analytical chemist? Our survey shows that the most common tasks (a maximum of 3 answers was allowed) among all the responding analytical chemists were the following: method development, project management, routine analysis, and teaching (see Fig. 4). Analytical chemists in industry responded with method development, routine analysis, and project management as their primary work tasks, while those in academia have a larger focus on teaching, fundamental research and applying for research grants. For university personnel, teaching usually took 11–30% of their total work hours.

Responders were also allowed to describe their work tasks in free-text, and a typical answer from academic staff were “*Supervision of doctoral students, teaching, project management, and various administrative assignments*”, and “*Course responsibility, lectures and lab course development*”. There was a larger variation in answers from industry responders, which for instance included “*Trouble-shooting, method development, method validation, method transfer, training of employees, and purchase of instruments*”; “*Service and repair of analytical instruments*”; “*Quality assurance of medicines from a lifespan perspective*”; “*Method development and routine analysis. Technical support for other chemists.*”; and “*Sell, install and support laboratory instruments*”.

**Fig. 5:**
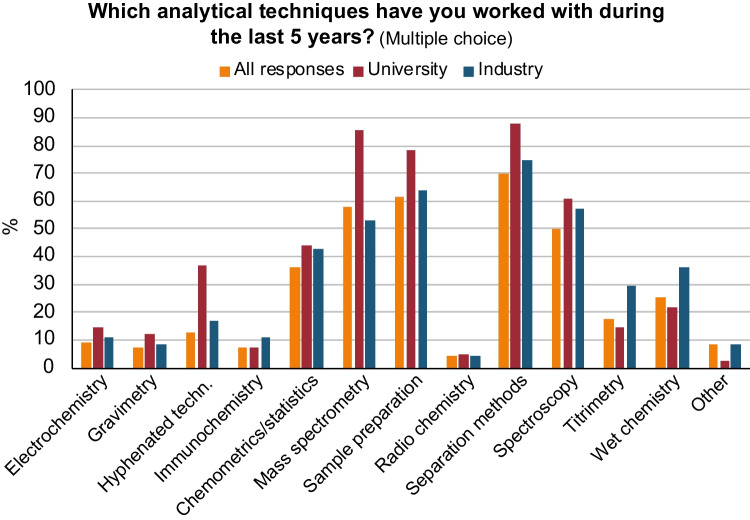
Responses to the question: “Which analytical techniques have your worked with during the last 5 years?”. “All responses” include persons from industry, university, research institutes, authorities, students, and retired. On the *y*-axis: response in percentage from each sub-category

**Fig. 6:**
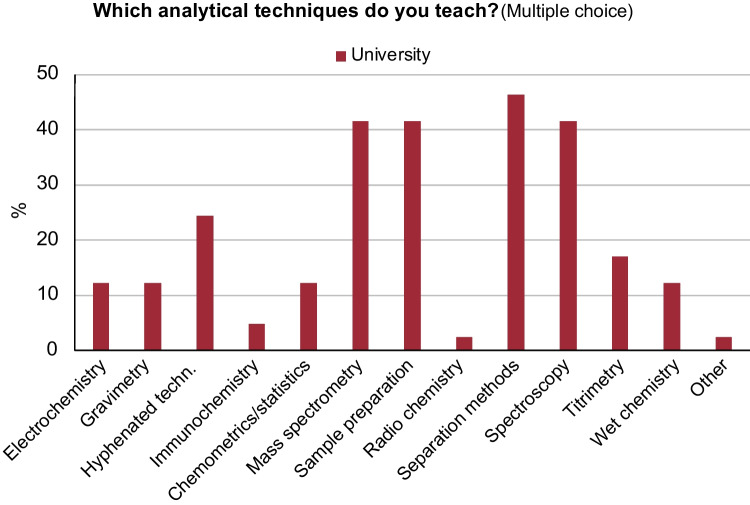
Responses to the question: “Which analytical techniques do you teach?”. The question was only asked to persons at the university who are currently teaching. On the *y*-axis: response in percentage from the sub-category “University”

When it comes to the question of which analytical techniques are being used in university, industry, and in general, the answers are fairly similar. Most analytical chemists have worked with separation methods, sample preparation, mass spectrometry, and spectroscopy during the last 5 years (Fig. 5). For university staff that are involved in teaching, the most commonly taught techniques were the same as those most commonly being used in work tasks, i.e., separation methods, sample preparation, mass spectrometry, and spectroscopy (Fig. 6). Hyphenation between techniques was also an important topic. This correlation between teaching and real-world usage implies that the planning of the teaching is well grounded in the work that analytical chemists perform.

About half of the analytical chemists employed at universities are project leaders, while only one industry respondent is a research leader. At the university, the research groups vary considerably both in size and composition, but consist mainly of PhD students and postdocs, although master students, bachelor students, and researchers also are part of the constellations. The number of each category typically varies between 0 and 5 each per research group.

A majority (60%) of academics apply for research funds from larger and smaller Swedish funding organizations and from the European Union/European Research Council (EU/ERC), which is the third most frequent contributor. The question “How do you experience the funding agencies’ attitudes towards research in analytical chemistry?” was met with a large variation in free-text answers. Here follows two such examples: “*It is far easier to obtain research funding for applied research than for basic research, and easier with collaborations than single projects*”, and “*They wish it to be applied, i.e. a tool is to achieve goals, the tool itself is not the goal. It is hard to get funding for analytical chemistry on its own, without a directly coupled application of the technique*”.

## What is analytical chemistry of today and what is the role of an analytical chemist in society?

Analytical chemistry is a science that is directed towards expanding our scientific knowledge by improving chemical analysis methods, often as a response to new or increasing demands from society. The research area of analytical chemistry has undergone a large transformation over the last decades; from early instrument construction and basic methodology, to today’s focus on method development and applications of analytical chemistry in disciplines outside chemistry e.g., in biosciences [4]. Method development involves obtaining faster, simpler, more sensitive, more complex/informative, and/or more environmentally sustainable analytical methods. It can be purely driven by curiosity, but more often it is addressing a specific analytical issue or task to meet a societal need. The subject thus includes both basic and applied research. Access to relevant advanced instrumentation is often very important both for satisfactory study results and for the practical execution of research, regardless of specialization. Unfortunately, the requirement for advanced equipment often involves large costs due to both the procurement and maintenance of the equipment, which can cause problems, especially with a limited budget. Limited access to, or broken equipment, can cause delays in both education and research. In addition, as discussed by Kovarik et al. [5], teachers at the university are often expected to do all the maintenance and repair of analytical equipment in addition to their regular teaching. One possible way forward is to establish collaborations between research groups that have access to different advanced instrumentation. Another example is that several organisations, private as well as public, have established so called “Core facilities”, where expensive instrumentation and competencies are consolidated and available to the entire company or university. It should be emphasized, however, that advanced and well-working equipment neither guarantees nor is always necessary to obtain a high level of scientific achievements. Fundamental knowledge, in theory and in practice, must always form the foundation of which analytical chemistry is built upon.

The changing role of the analytical chemists has been discussed in the feature article by Adams and Adriaens [6], where it is described how the research field of analytical chemistry has gone through a metamorphosis, from previous “measurement science” to current “information science”. Classical analytical chemistry driven by metrology and quality assurance has evolved over the years into a field that makes an important impact in the “big data era”, driven by hypothesis generation and systemic/holistic approaches.

In another review by Miguel Valcárcel [7], the transformation of analytical chemistry over the last 50 years is discussed in the context of chemistry. Valcárel argues that the information-related component of chemistry is frequently missed. He proposes a rather comprehensive definition of analytical chemistry, thereby pointing out the need for analytical chemistry in many disciplines other than chemistry: “*Analytical chemistry can be defined as the chemical metrological discipline that develops (R&D), optimizes, and uses tools and measurement processes in order to strengthen its capabilities to extract information – particularly to obtain quality (bio)chemical information about objects and systems of natural/artificial nature in order to fulfil specific needs or requirements with a view to facilitating grounded, timely decisions in scientific, technological, economic, and social areas.*” [7].

### Is it valid to say that analytical chemistry is becoming mainly a tool for other disciplines?

Valcárcel communicates a relatively negative picture, i.e. “*Analytical chemistry is often viewed as a second-class discipline of chemistry despite the undeniable need for reliable (bio)chemical information in making grounded, timely decisions in many fields of action, such as environmental sustainability, healthcare, nutrition, agriculture, hygiene, transport, sports, dressing, culture, home, or building.*” [7] He also states that analytical chemists, chemists from other disciplines, as well as other professionals are all to blame for the distorted image of analytical chemistry. As an example, analytical chemists working in the fields of environmental monitoring or material development call themselves environmental chemist and material scientist, respectively, probably for simplicity, but it may contribute to obscuring the role of the analytical chemist.

Our survey shows that analytical chemists believe that professionals from other disciplines view analytical chemistry mainly as a support-science of lower status, and not as a stand-alone discipline. Free-text answers to the question “How do you think others (from other disciplines) view your role as analytical chemist?” give answers such as “*Sorry but low status*”, “*Someone who provide analytical data and develop test methods*”, “*Not highly appreciated, but good they are available, but slow in their work*”, “*As a technical operator that supports other disciplines*”, and “*At best as a helpful, and supporting, science. Not as a stand-alone discipline.*” However, when we ask chemists from other disciplines, the picture is somewhat more positive, e.g., giving free-text answers such as “*Important! It develops measurement techniques*”, “*Provide other chemists (and scientists/engineers) with good data*”, “*Supporting science*”, “*Repetitive*”, “*Picky*”, and “*Creating tools for efficient analysis of environmental samples, tracing leaks, surveying drug metabolization or mapping distributions of compounds *etc*., is extremely important for all parts of society*.”

**Fig. 7:**
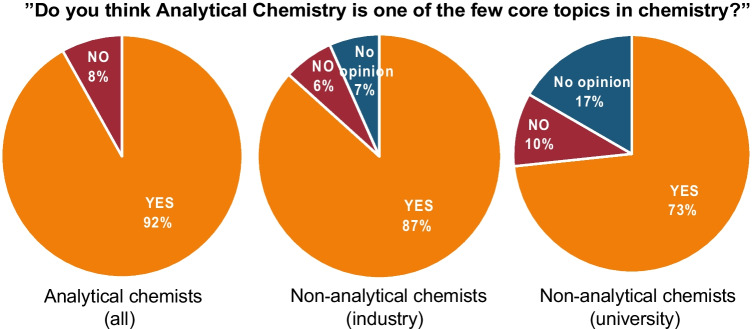
Responses to the question “*Do you think analytical chemistry is one of the few core topics in chemistry?*” Question asked in the second survey to analytical chemists (49 responses, left figure), Survey to non-analytical chemists in industry (30 responses, centre figure), and Survey to non-analytical chemists at universities (30 responses, right figure)

Analytical chemistry is generally considered to be one of the few core subjects within chemistry (see Fig. 7). However, it seems that our self-image is somewhat distorted, and that it is necessary for us to support each other with a more appropriate definition of analytical chemistry and protect the subject’s natural role in collaborative projects. It is important that the evolution of analytical chemistry is discussed among professionals both within the discipline and outside, so that it can adapt to future needs in the society. In such discussions, it is imperative to not only consider the transformation of fundamental and applied research in analytical chemistry, but also the industry perspective in terms of needed competence in chemical analysis, of which the latter strongly connects to the education.

### So, what is the role of analytical chemists in the society?

In our survey, free-text answers from analytical chemists answering the question “How do you view the role of analytical chemists in the society?” show a large variety of suggestions. From university responders, we read for instance: “*An analytical scientist is more concerned about the quality of the measurements*”, “*Contributes with knowledge and support for accurate measurements, enhances the competence of instrument operators performing chemical measurements, and improves/invents new analytical methods and techniques*”, “*An important chemistry discipline, which gives a holistic view of different challenges in the society, from the importance of proper design and data points to the ability to draw certain conclusions depending on how analysis was done. More and more challenges requiring complex chemical analysis demands a continuous development of the field, in addition to being an important support (“tool”) for other disciplines*”, “*It is a field of high impact for society and also towards a sustainable development of the society*”, “*As a discipline that supplies chemical knowledge that is sceptical and scientific. It relates such important knowledge as dosage and sensitivity matters, and further a critical view on what can be measured and not*”, and “*Essential in all fields dealing with manufacturing, research and health*.”

From industry responders, we read the following: “*To develop technologies and methods for chemical measurements, work to provide data from chemical measurements, and share knowledge of analytical chemistry*”, “*Essential for many industrial processes, personal care, and health*”, “*Someone who work to provide chemical data, by developing and performing analytical testing, or in other ways support or lead activities related to chemical measurements and can explain and assess how chemical measurements are performed and their underlying principles*”, “*As a guarantee of quality of results and research*”, and “*Analytical chemists are essential to establish safe products and testing of products*.”

**Fig. 8:**
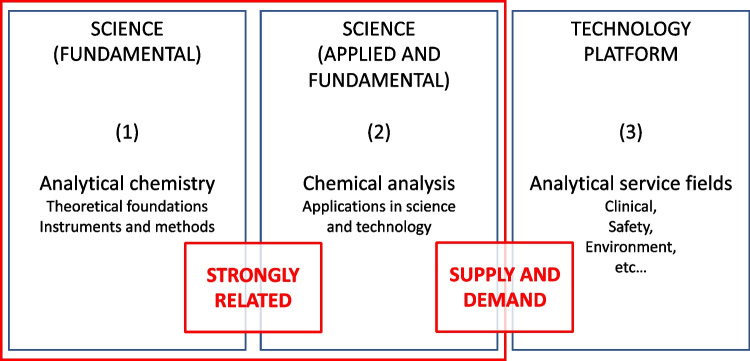
The anatomy of analytical chemistry: (1) analytical chemistry, (2) chemical analysis, and (3) analytical services. Cited from [6]

Adams and Adriaens discuss in their review article [6] the role of analytical chemists in society and how this has changed over the last several decades. Figure 8 illustrates the three main roles of analytical chemists in society: (i) fundamental science (analytical chemistry); (ii) applications in science and technology (chemical analysis); and (iii) analytical service (technology platform), of which fundamental and applied science are strongly related and overlapping. All these roles are undoubtedly of importance to society. In universities, the fundamental science aspect is more pronounced, while industry has proportionally more analytical service activities.

## Does our education give analytical chemists the right knowledge and skills?

### How easy is it to get a job as an analytical chemist?

There are several published surveys that have been conducted with a focus on the job market situation for chemists in Europe, e.g. [1] that clearly demonstrate that analytical chemists largely do not have trouble finding employment. Considering all areas of chemistry, training in analytical chemistry, organic chemistry, and chemical engineering are the three most desired educational backgrounds for recruiting companies.

### What is the recruitment situation?

**Fig. 9:**
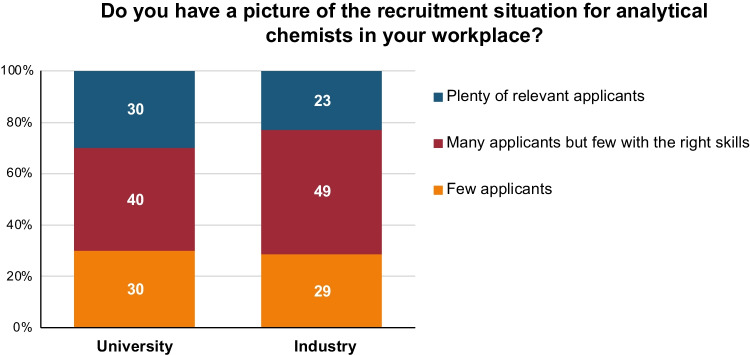
Responses to the question: “Do you have a picture of the recruitment situation for analytical chemists in your workplace?”. Responses include persons from university (left) and industry (right). On the *y*-axis: response in percentage from each sub-category

In our survey, we asked analytical chemists from both university and industry about the recruitment situation for analytical chemists in their workplace (Fig. 9). In industry, 77% of the responders answered either “few applicants” or “many applicants but few with the right skills”. In university, the corresponding number was 70%. Clearly, either not enough analytical chemists are being educated, or they are not taught the right skills.

### What knowledge and skills are required?

The expectations from analytical chemists in industry are typically different from expectations in academia. The survey showed e.g. that the ability to speak and write in English, a basic degree in analytical chemistry, and the “overall analytical chemistry thinking” were ranked as the most important skills when recruiting analytical chemists at the university, while industry considered the “overall analytical chemistry thinking” and experience in project management as the two most important aspects. Unfortunately, the number of respondents were very low from industry for this particular question, most likely due to the low number of persons in industry being involved in recruitment processes.

**Fig. 10:**
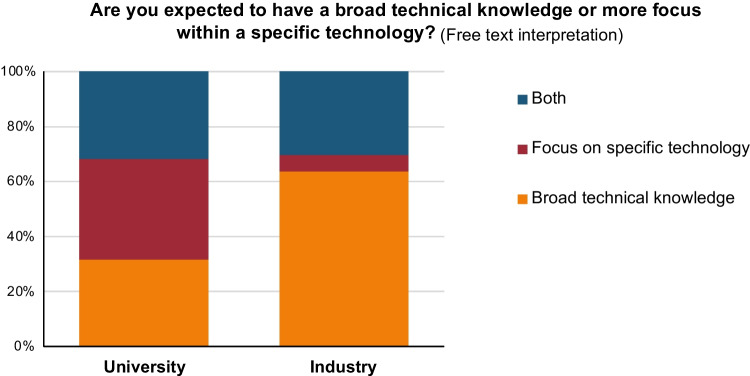
Responses to the question: “Are you expected to have a broad technical knowledge or more focus within a specific technology?”. Responses include persons from university (left) and industry (right). On the *y*-axis: response in percentage from each sub-category. Based on interpretation of free-text answers. Non-concluding answers were removed

There were clear differences regarding expectations on technical knowledge in our survey (see Fig. 10). On the survey question “Are you expected to have a broad technical knowledge or more focus within a specific technology?”, 64% of the industry respondents answered that they were expected to have a broad technical knowledge in analytical chemistry, while only 6% answered “focus on specific technology”. Among responders from the universities, the numbers were divided relatively equally between “broad technical knowledge”, “focus on specific technology”, and “both”, respectively.

Some of these differences also show up in the data on the most common work tasks as presented in Fig. 4, where industry responders do more “routine analysis” and “other” in comparison to university responders. We did not specifically ask about quality work, of which many analytical chemists are both involved in and responsible for in industry. Competence in quality management (from method validation to quality systems) may be another way for analytical chemists to find occupation in fields that are not strictly chemistry-related, e.g., within product or production development.

The higher demand for specialization in the universities is most likely a result of the strive to do fundamental research (see for instance in Fig. 4 — fundamental research is three times more common as a work task in university compared to in industry). The need for broad technical competence among industry staff is most likely explained by problem-solving issues concerning process development, product assessment, environment, etc., challenges that should be resolved efficiently with the analytical chemistry staff at hand.

### Does our education give analytical chemists the right knowledge and skills?

**Fig. 11:**
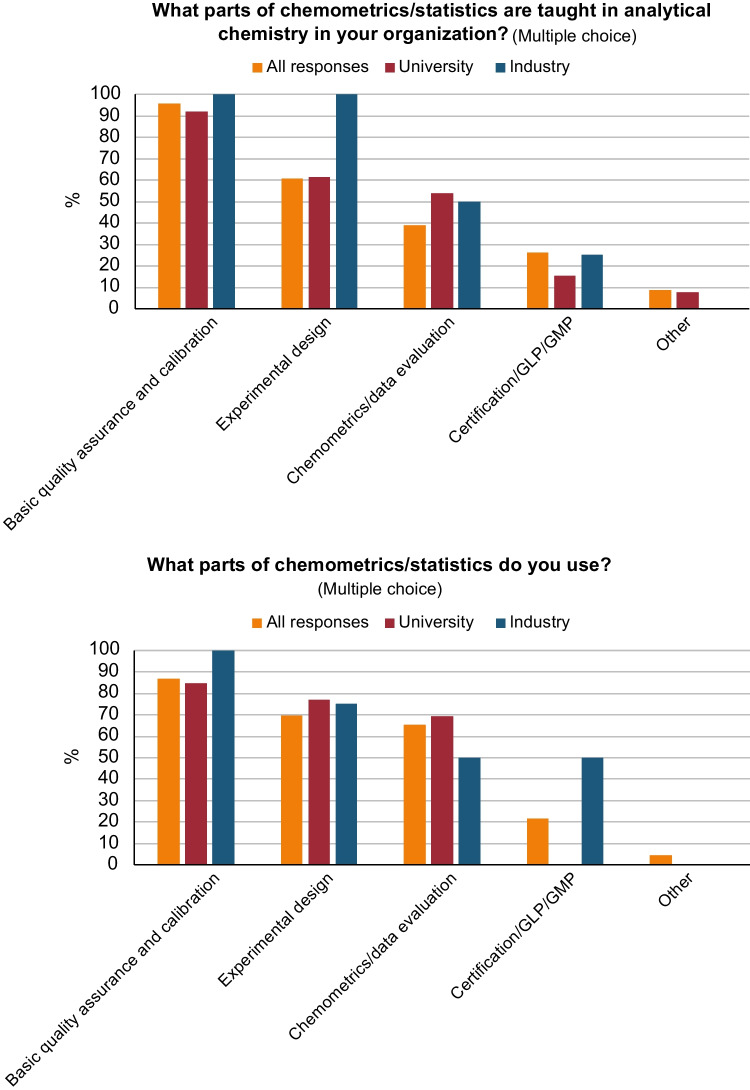
Responses to the questions: “*What parts of chemometrics/statistics are taught in analytical chemistry in your organization?*” (top), and “*What parts of chemometrics/statistics do you use?*” (bottom). “*All responses*” include persons from industry, university, research institutes, authorities, students, and retired, in total 23 responses. University, 13 responses, and Industry, 4 responses. On the *y*-axis: response in percentage from each sub-category

In our survey, we observe that when responders were asked which analytical techniques they use (Fig. 5), it corresponds well with the analytical techniques that are taught at the university (Fig. 6). The only exception is chemometrics and statistics, which is used to a greater extent (> 40% in Fig. 5) than it is being taught (ca 12% in Fig. 6). It is for this reason that we asked the questions “What parts of chemometrics/statistics are taught in analytical chemistry in your organization?” and “What parts of chemometrics/statistics do you use?” in our second survey (see Fig. 11).

From the above outcome, it seems that industry sees the same transformation of the field of analytical chemistry as discussed above (Adams and Adriens [6], from measurement science to information science), even though there is more of measurement science still in industry. However, industry is training people in chemometrics/statistics and is performing chemometrics/statistics, though not always in the same areas as academics. The remaining question, based on the data, is whether students are being trained to perform chemometrics/statistical analysis to meet the need of industry. This should be investigated further since it is important that education in analytical chemistry is well adapted to current needs, especially in industry.

## Conclusions and outlook

Analytical chemists work in many different environments and with a large variety of work tasks. The primary difference between the work tasks done by analytical chemists in university and in industry are teaching and fundamental research, which is mostly found in university, and routine analysis, which is mostly found in industry. The analytical techniques we teach our students generally correspond well with those that are used in both industry and university. An exception is chemometrics, which is used to a larger extent that it is taught.

Analytical chemistry is regarded as one of the few core subjects within chemistry, both by analytical chemists and by non-analytical chemists, but for the latter group it is especially the industry-affiliated chemists that consider analytical chemistry a core subject within chemistry. Analytical chemists describe their role in society quite broadly, with answers ranging from the more conventional role of method development and quality assurance to the more holistic view of analytical chemistry as having an important role in solving different challenges in society. However, analytical chemists have a largely negative view on how they believe other disciplines view the role of an analytical chemist in society, best summarized as someone developing analysis methods and providing analytical data. This self-image is only partly true, and our survey demonstrate that other disciplines can see the importance of analytical chemists in all parts of society.

Our survey also shows that a vast majority of analytical chemists that work with recruitment experience a situation of “few applicants” or “many applicants but few with the right skills”. Based on our survey, each of about ten universities graduate 1–20 MSc students in analytical chemistry annually. This number is too low to satisfy the need for analytical chemists in industry. One question that needs further investigation is whether we educate analytical chemists with the right knowledge and skills, especially considering the transformation of the subject of analytical chemistry during the last couple of decades. Should we introduce a more holistic view in our educations, thereby preparing our students for more cross-disciplinary interactions, or should we continue with the classical “linear” view of analytical chemistry as a measurement science? A deeper follow-up study with interviews with analytical chemists and other key personnel in industry would be necessary to answer this question. After all, analytical chemistry education should follow Murray’s definition: “Analytical chemistry is what analytical chemists do”. And since “what analytical chemists do” is something that changes with time, it is also necessary for our education to reflect this change.

In summary, it is clear that analytical chemistry will remain as an important part of society in the future, as discussed by Bergquist & Turner in [2] — “The future of analytical chemistry can be found in cross-disciplinary and interdisciplinary areas, focused on solving important societal challenges and engaging young and bright scientists.” In fact, analytical chemistry goes even beyond chemistry, bridging disciplines such as environmental science, biology, and medicine.
